# Microwave Gas Sensors Based on Electrodeposited Polypyrrole–Nickel Phthalocyanine Hybrid Films

**DOI:** 10.3390/s23125550

**Published:** 2023-06-13

**Authors:** Ileana-Alexandra Pavel, Alexis Lasserre, Léo Simon, Jérôme Rossignol, Sophie Lakard, Didier Stuerga, Boris Lakard

**Affiliations:** 1 UTINAM—UMR CNRS 6213, Université de Franche-Comté, 25030 Besançon, France; ileana-alexandra.pavel-licsandru@univ-fcomte.fr (I.-A.P.); slakard@univ-fcomte.fr (S.L.); boris.lakard@univ-fcomte.fr (B.L.); 2 GERM, Laboratoire Interdisciplinaire Carnot de Bourgogne, Department Interfaces, UMR CNRS 6303, UB, 21078 Dijon, France; alexis.lasserre@u-bourgogne.fr (A.L.); leo_simon01@etu.u-bourgogne.fr (L.S.); didier.stuerga@u-bourgogne.fr (D.S.)

**Keywords:** conducting polymers, phthalocyanine, microwave transduction, gas sensor, ammonia

## Abstract

Previous studies have shown that the incorporation of sulfonated metallophthalocyanines into sensitive sensor materials can improve electron transfer and thus species detection. Herein, we propose a simple and easy alternative to the use of generally expensive sulfonated phthalocyanines by electropolymerizing polypyrrole together with nickel phthalocyanine in the presence of an anionic surfactant. The addition of the surfactant not only helps the incorporation of the water-insoluble pigment into the polypyrrole film, but the obtained structure has increased hydrophobicity, which is a key property for developing efficient gas sensors with low sensitivity to water. The obtained results show the effectiveness of the materials tested for the detection of ammonia in the range of 100 to 400 ppm. It is shown by comparing the microwave sensor responses that the film without nickel phthalocyanine (hydrophilic) produces greater variations than the film with nickel phthalocyanine (hydrophobic). These results are consistent with the expected results since the hydrophobic film is not very sensitive to residual ambient water and therefore does not interfere with the microwave response. However, although this excess response is usually a handicap, as it is a source of drift, in these experiments the microwave response shows great stability in both cases.

## 1. Introduction

Constant environmental monitoring and pollution control have become imperative, as environmental pollution has tremendously increased due to rapid modernization and industrialization. More and more, the detection of harmful gases has become an important topic, not only for the assessment of pollutants in the atmosphere but also for human health as a non-invasive clinical diagnostic tool. Gaseous NH_3_ in high concentrations can lead to skin and eye irritation, the destruction of the respiratory tract mucosa, and even death [1,2]. On the other hand, in small quantities (400–14,700 ppb), ammonia is present in the breath of patients suffering from hepatic encephalopathy [3,4], end-stage renal disease [5,6], halitosis, or helicobacter pylori infection [7,8].

On one hand, conducting polymers are suitable candidates for sensors as their chemical, electrical, and structural properties can be easily modulated by adding new moieties to the monomer or introducing different dopants [9,10]. For example, the electrical conductivity of poly(N-methylaniline) and polypyrrole can be increased by the addition of an anionic surfactant [11,12], and its adhesion can be improved by dopamine functionalization [13]. Thus, electrochemical sensors based on polypyrrole/SDS films were used to detect p-nitrophenol and iron (II) cations [14,15]. On the other hand, the presence of phthalocyanine macrocycles in a coating generally favors electron transfers between the sensor and the analytes, as they are electron donors, thus offering the ability to interact selectively with different analytes due to their cavity structure [16,17].

Therefore, it seems interesting to combine the remarkable properties of conducting polymers and phthalocyanines by incorporating a phthalocyanine into a conducting polymer matrix. However, this incorporation is not easy due to the low solubility of phthalocyanines in aqueous solution; therefore, it is often necessary to use sulfonated phthalocyanine [18,19]. For example, a polypyrrole-doped cobalt-tetrasulfonated phthalocyanine film was electrodeposited on a carbon-fiber ultramicroelectrode and used to perform the amperometric detection of 2-mercaptoethanol by differential pulse voltammetry [20]. The addition of tetrasulfonated nickel phthalocyanine to an acidic solution of aniline resulted in an accelerated deposition of polyaniline, and the resulting composite film exhibited catalytic activity toward the electro-oxidation of ascorbic acid in a nearly neutral buffer [21]. Tetrasulfonated copper phthalocyanine [22] and tetrasulfonated nickel phthalocyanine [23] were incorporated into polyaniline films, which both demonstrated high sensitivity to ammonia. Similarly, the incorporation of a sulfonated cobalt phthalocyanine into a polypyrrole film during electropolymerization resulted in a remarkable improvement in the response to ammonia, which was found to be fast, reproducible, and very sensitive [24]. The NO_2_-sensing capability of polypyrrole was also enhanced after it was functionalized with iron (III) phthalocyanine-4,4′,4″,4‴-tetrasulfonic acid monosodium salt since the resistance of the film decreased strongly during exposure to NO_2_ gas at room temperature [25]. To further facilitate the incorporation of a cobalt-tetrasulfonated phthalocyanine inside a conducting polypyrrole film during its electropolymerization, Muthuraman et al. added an anionic, cationic, or neutral surfactant within the electrolyte solution [26]. The physico-chemical analyses confirmed the orientation of the film variation according to the surfactant type and identified the immobilization site of the metallophthalocyanine. Moreover, it was shown that the morphology and conductivity of the films were strongly impacted by the presence and nature of the surfactant. In another study, Tiwari et al. added a cationic surfactant to the electrolytic solution to facilitate the incorporation of a non-sulfonated copper phthalocyanine into a conducting polypyrrole film. This film was then successfully used for the selective detection of the nerve gas simulant dimethyl methyl phosphonate [27].

**Scheme 1:**
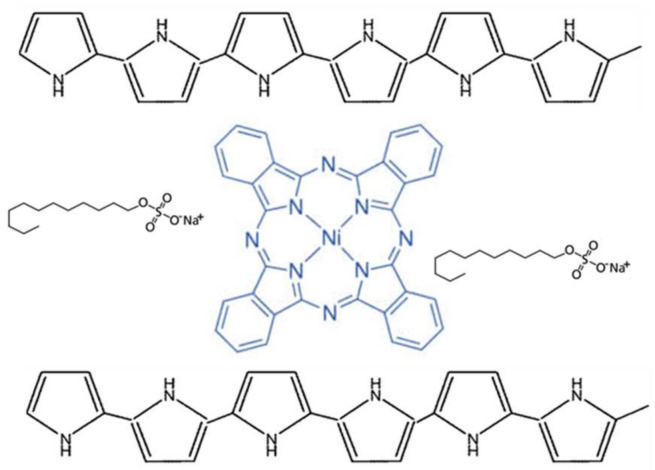
Schematic representation of polypyrrole with nickel phthalocyanine in the presence of an SDS surfactant.

Based on this work, the present study aimed to develop a less expensive alternative to the use of sulfonated phthalocyanines. Our approach was to incorporate a non-functionalized nickel phthalocyanine into a polypyrrole film using a sodium dodecyl sulfate (SDS) anionic surfactant during electropolymerization (Scheme 1). The physico-chemical properties (morphological features and wettability) of the films obtained with and without metallophthalocyanines were compared. These polypyrrole/phthalocyanine composite films, as well as polypyrrole films, were then tested as sensing layers of microwave gas sensors for the detection of ammonia, using their ability to operate at room temperature, contrary to metal oxide-based chemiresistors that typically operate at 300–500 °C [28,29]. Microwave sensors are a relatively new type of sensors that are characterized by a propagative structure (e.g., a microwave planar resonator) surrounded by a sensing film [30]. The sensing principle is based on the propagation and reflection of electromagnetic waves into the sensitive material. Thus, the sensor measures the frequency evolution of the sensitive material’s permittivity after the adsorption of gas molecules on the surface of the sensitive layer at room temperature [31]. Among the materials already used as sensitive layers in microwave sensors for gas detection, metal oxides [32], zeolites [33], conducting polymers [34], and phthalocyanines [35] appear to be promising materials. Through this study, the aim was to determine if the composite films obtained by the association of a conductive polymer and a metallophthalocyanine can also lead to highly sensitive responses to ammonia via microwave transduction.

## 2. Experimental Section

- Materials

Pyrrole (Py), sodium dodecyl sulfate (SDS), and nickel phthalocyanine (NiPc) were reagent-grade and were used as received from Sigma Aldrich. All the solutions were prepared in MiliQ water. Py and SDS were prepared at a concentration of 0.1 M, and NiPc was prepared at 0.001 M. The solvent used for the electrodeposition experiments was double-distilled water.

- Electrochemistry

All the electrochemical experiments were performed using a SPELEC potentiostat/galvanostat controlled by a PC via a DropView software interface. The electrochemical cell consisted of a classical three-electrode setting with a Saturated Calomel Electrode (SCE) reference electrode, a platinum plate (5 × 5 × 0.1 cm) as the counter electrode, and a platinum wire (0.785 mm^2^) or fluorine-doped tin oxide (FTO) substrate (R = 80 Ω/square, 10 mm × 30 mm) as the working electrode.

- Mechanical profilometry

The thickness and roughness of the films were measured using a Dektak 150 Surface Profiler from Veeco. The arithmetic roughness (Ra) was measured with a scan length of 5000 µm at a scan speed of 50 µm/s. The thickness (T) was measured using the same operating conditions after the creation of a physical sharp step in the film. The reported Ra and T values are the averages of at least 3 measurements performed at different locations in the films.

- Scanning electron microscopy (SEM)

The surface topography of the films was imaged, without prior metallization, using a high-resolution Thermo Scientific Apreo 2 Scanning Electron Microscope. The electron beam energy used was 5 keV, and the working distance was 10 mm.

- Energy-dispersive X-ray spectroscopy (EDS)

Energy-dispersive X-ray spectroscopy was performed on a FEG TESCAN scanning electron microscope with an EDS detector. The EDS quantification was obtained at 7 kV and 1.1 mA using Idfix software from SamX with an XPP matrix correction (ZAF improvement).

- Water contact angle measurements

The wettability of the films was studied using a contact angle analyzer (Digidrop from GBX) equipped with a CCD camera, a sample stage, and a syringe holder. A 5 µL drop of ultrapure water was formed at the tip of a syringe needle and placed onto the sample surface by raising the sample until contact was made. Then, an image of the drop was captured, and the contact angle was determined by drawing the tangent close to the edge of the droplet. At least five drops were deposited on each film.

- Infrared spectroscopy

The infrared spectra of the films were obtained using Infrared Reflection Absorption Spectroscopy (IRRAS) in reflection geometry at a grazing-incidence angle of 65°. The infrared spectrometer used was a Vertex 70 FT-IR equipped with a DGTS detector. For each film, 256 scans were recorded with a 2 cm^−^^1^ resolution.

## 3. Results and Discussion

### 3.1. Electrodeposition and Characterization of PPy-SDS and PPy/NiPc-SDS Films

- Electrochemical deposition of PPy-SDS and PPy/NiPc-SDS films

**Figure 1:**
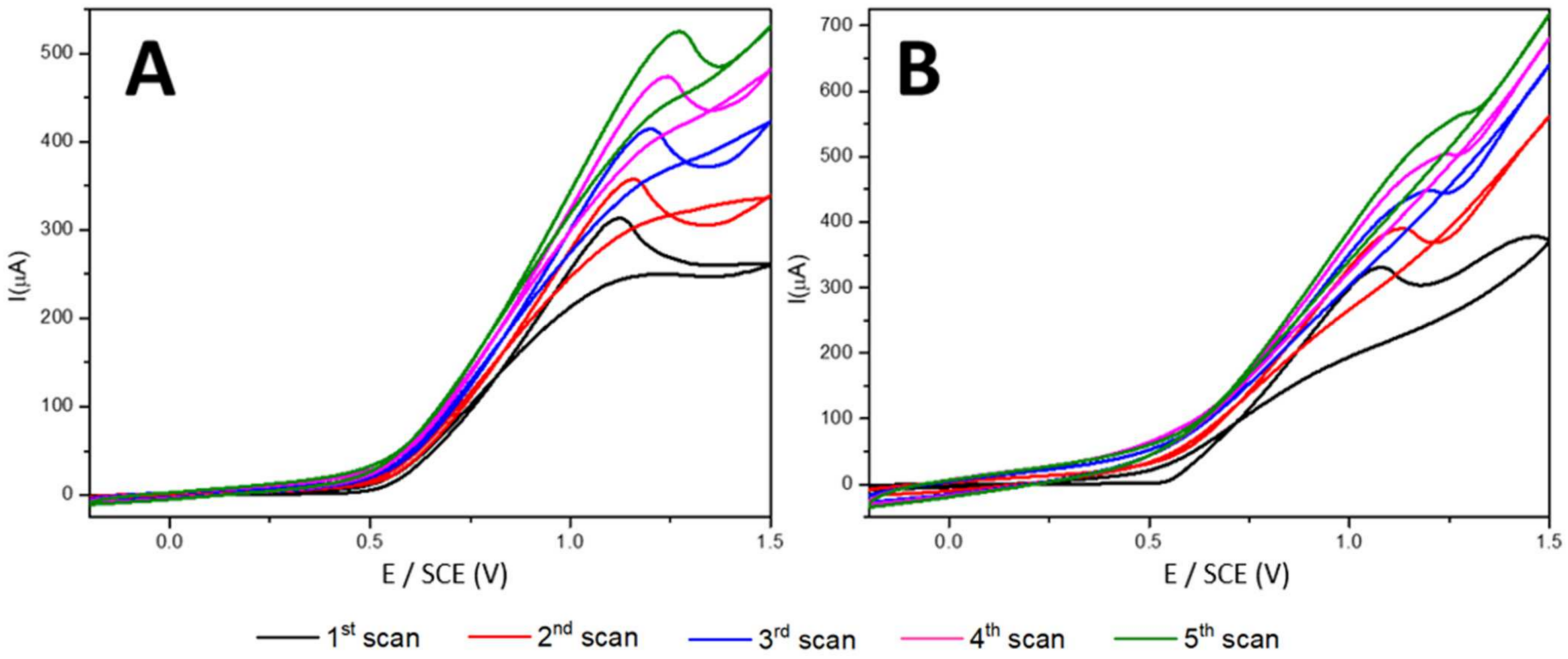
(**A**) Cyclic voltammograms for the electropolymerization of PPy films on a Pt electrode in the presence of SDS. (**B**) Cyclic voltammograms for the electropolymerization of PPy films on a Pt electrode in the presence of SDS and NiPc.

First, two polymer films were formed using cyclic voltammetry (CV) from an aqueous solution of SDS in the presence (PPy/NiPc-SDS) or absence of nickel phthalocyanine (PPy-SDS). To form these conductive films, five potential scans were performed from −0.2 to +1.5 V/SCE at a scan rate of 50 mV/s at a platinum working electrode. The cyclic voltammogram recorded during the potentiodynamic growth of polypyrrole in the presence of SDS (Figure 1A) displayed an increase in current and a shift in the oxidation pic to higher values as the number of cycles increased due to the growth of PPy films and the incorporation of the SDS anions acting as dopants. The intensity of the oxidation peaks was between 300 and 500 µA, which was much lower than the values obtained during the polymerization of pyrrole in water in the presence of LiClO_4_, which were on the order of several µA [21,22,23,25,26,36,37,38]. Indeed, during the oxidation of pyrrole, the PPy film grows and becomes positively charged so that the negative polar heads of SDS anions can attach to the polymer backbone and occupy a large place that can then no longer be occupied by the pyrrole units [22,23,24,25,26,36,37,38]. On the contrary, in the absence of SDS anions and in the presence of small perchlorate anions, this steric hindrance does not exist. Thus, perchlorate anions can strongly penetrate and dope the PPy backbone, whereas SDS anions lead to a less important doping, which results in a lower electrochemical activity for the deposits realized here than for PPy-LiClO_4_ films that were previously reported [22,23,24,25,26,36,37,38]. However, the presence of SDS also has advantages. Indeed, PPy-SDS films are thinner (1 µm), smoother (72 nm), and more homogeneous than PPy-LiClO_4_ electrodeposited in the same conditions, which are about 30 µm thick and 3 µm rough [21,22,23,25,26,38,39,40]. The cyclic voltammogram recorded during the potentiodynamic growth of polypyrrole in the presence of SDS and phthalocyanines (Figure 1B) displayed the same general trends since an increase in current and a shift in the oxidation pic to higher values as the number of cycles increased were also observed. The fact that the oxidation current increases with the number of cycles evidences the conductive character of the two electrodeposited polymer films.

**Figure 2:**
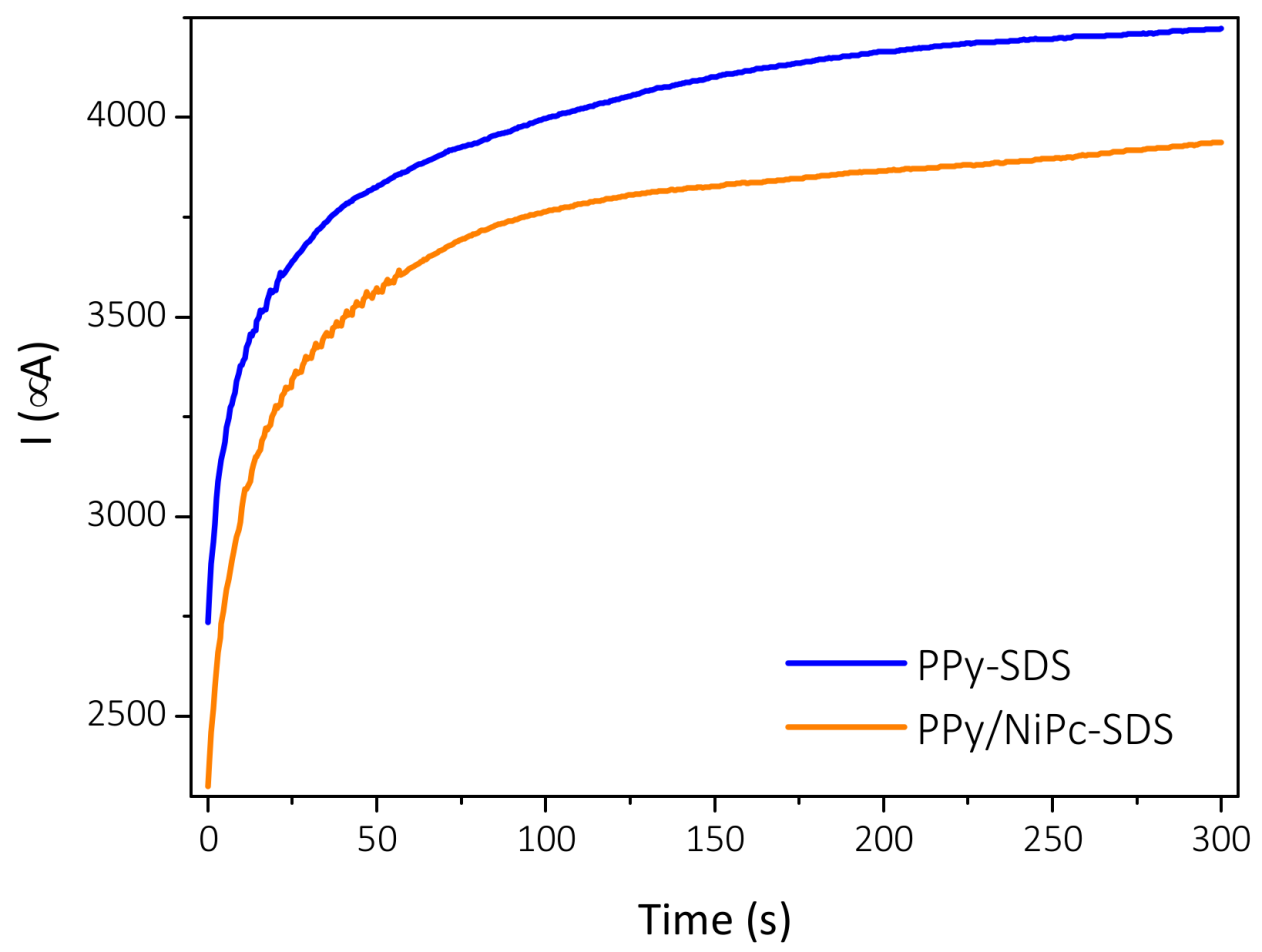
Amperometric polymerization of PPy-SDS and PPy/NiPc-SDS films on an FTO electrode.

Given the CVs that were obtained, it was decided to perform the electrodeposition of the PPy-SDS and PPy/NiPc-SDS films using chronoamperometry by applying a potential of +1.0 V/SCE, corresponding to the potential of the oxidation peak of pyrrole, for 5 min. This time was chosen for both experiments to obtain thick PPy films allowing for a better comparison of the properties of the films. Figure 2 displays the oxidation of the solutions on an FTO electrode. It can be observed that the curves have similar shapes, indicating that the electropolymerization process of the conductive films is not significantly different in the presence of nickel phthalocyanine (less than a 5% difference between the two curves).

- Characterization of PPy-SDS and Ppy/NiPc-SDS films

**Figure 3:**
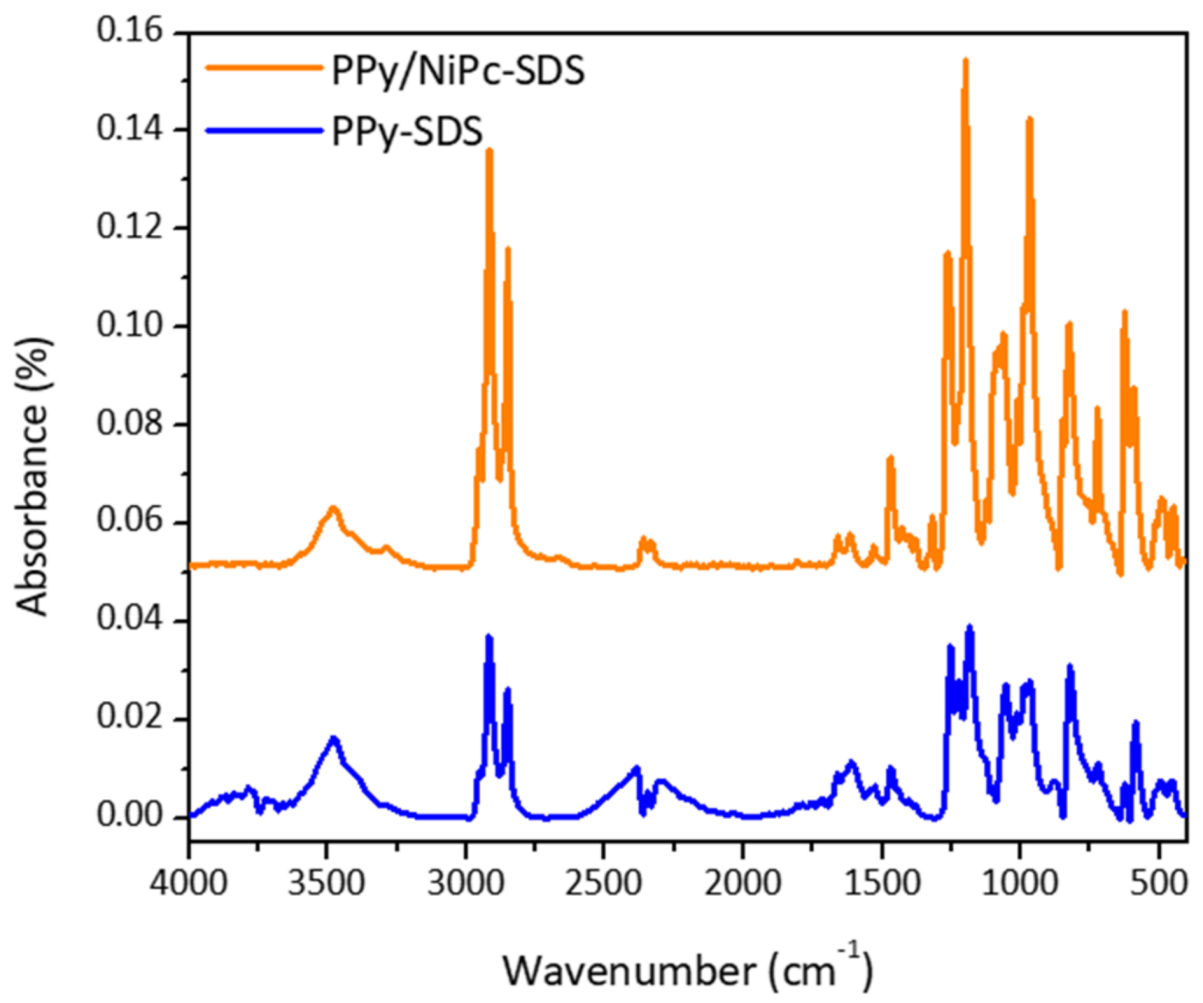
IRRAS spectra of PPy−SDS and PPy/NiPc−SDS films electrodeposited on an FTO electrode using chronoamperometry at +1 V/SCE.

**Figure 4:**
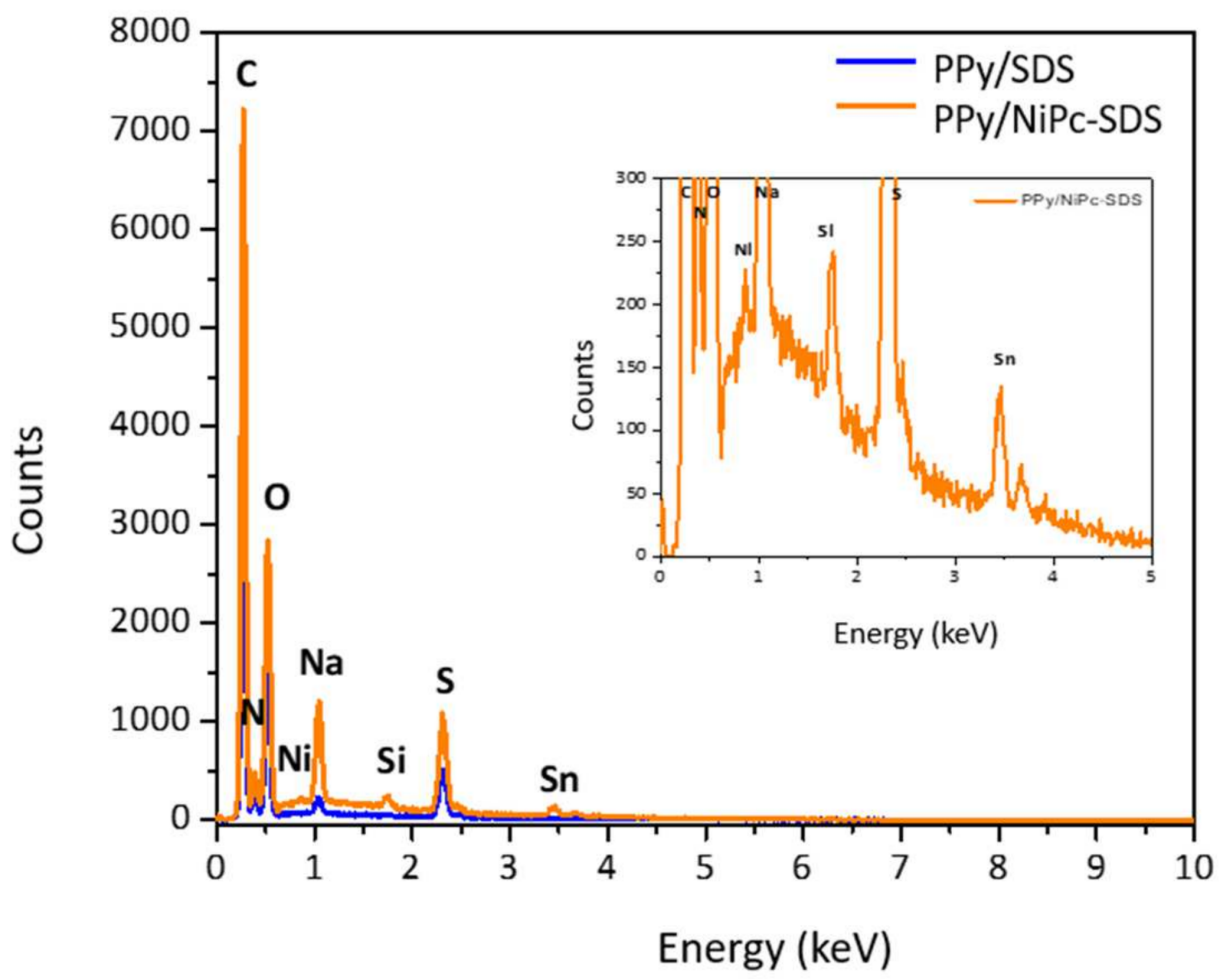
Energy-dispersive spectroscopy of PPy−SDS and PPy/NiPc−SDS films electrodeposited on an FTO electrode using chronoamperometry at +1 V/SCE.

The spectra of the PPy-SDS and PPy/NiPc-SDS films obtained using IRRAS spectroscopy (Figure 3) are in good agreement with those reported in the literature for polypyrrole [41,42,43]. The bands at 3400–3500 cm^−1^ are due to N–H stretching vibration, those below 3000 cm^−1^ are due to C–H stretching vibration, those at 1550 and 1480 cm^−1^ are due to the vibrations of the polypyrrole ring, those at 1250 and 1050 cm^−1^ are due to =C-H in-plane vibrations, and that at 1200 cm^−1^ is due to C-N stretching vibrations. Therefore, the infrared spectra confirm that the electrodeposited films possess the expected chemical structure. To analyze the incorporation of the NiPc into the polymer film, an energy-dispersive spectroscopy analysis was performed on both films (Figure 4). As presented in Table 1, the presence of the metallophthalocyanine was confirmed by the presence of nickel in the sample, and the presence of the SDS surfactant was confirmed by the presence of sulfur. Moreover, the FTO glass electrode contained the elements Si and Sn, which explains their presence in the EDS spectrum and shows that the analysis of the PPy/NiPc-SDS film was performed on the whole film and not only its surface. More precisely, the presence of the different elements was evidenced by the presence of peaks on the spectrum at 0.25 keV for carbon, 0.40 keV for nitrogen, 0.85 keV for nickel, 1.05 keV for sodium, 1.75 keV for silicon, 2.30 keV for sulfur, and 3.45 keV for tin atoms [44].

**Figure 5:**
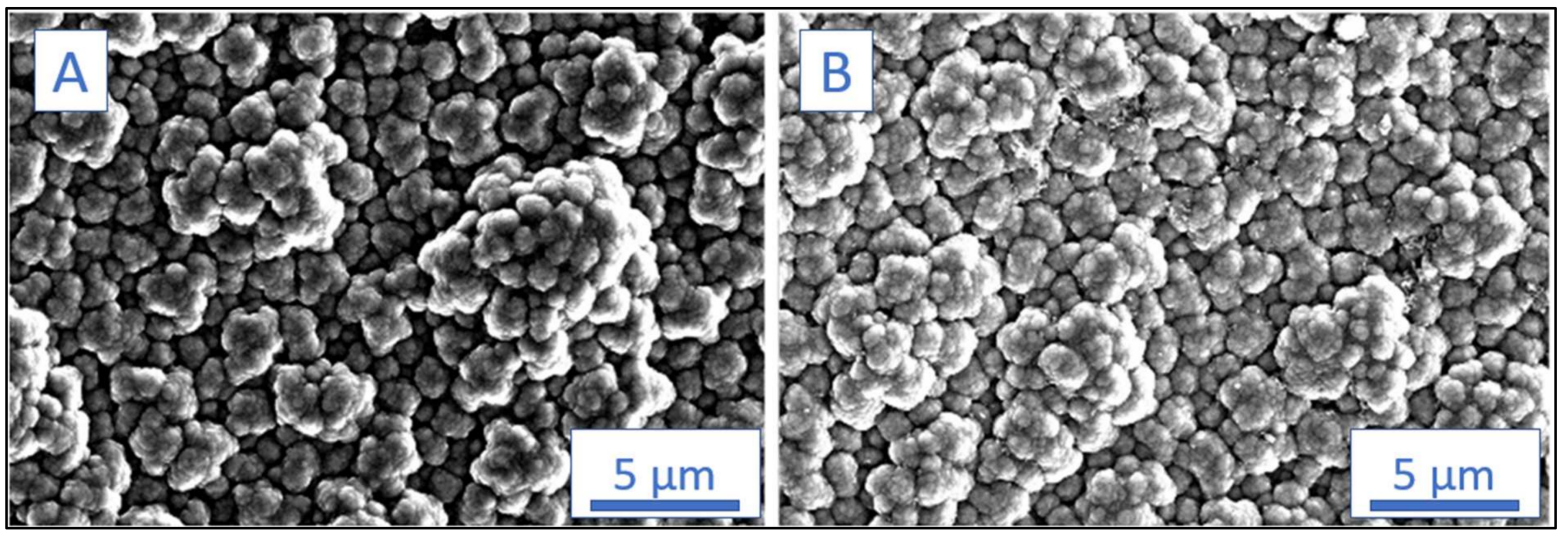
(**A**) SEM micrograph of PPy-SDS film electrodeposited on an FTO electrode using chronoamperometry at +1 V/SCE. (**B**) SEM micrograph of PPy/NiPc-SDS film electrodeposited on an FTO electrode using chronoamperometry at +1 V/SCE.

SEM images of the polypyrrole film obtained in the presence of the SDS surfactant exhibited a cauliflower structure that is characteristic of electrodeposited polypyrrole films (Figure 5A) [39]. The morphology of the electrodeposited film was not drastically affected by the incorporation of nickel phthalocyanine since the same cauliflower structure was observed for the PPy/NiPc-SDS film (Figure 5B). The thickness of the PPy-SDS film was 1 µm, while the thickness of the PPy/NiPc-SDS film was 3.5 µm, and the films had roughness measurements of 72 nm and 46 nm, respectively.

**Figure 6:**
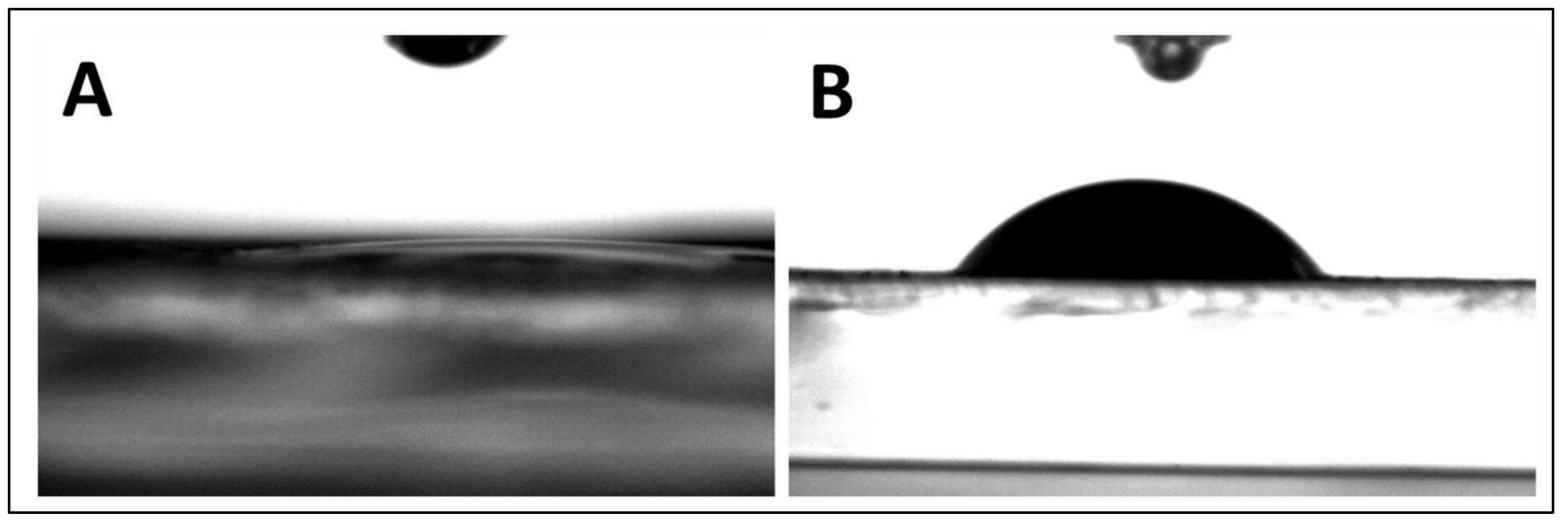
(**A**) Contact angle of PPy-SDS film electrodeposited on an FTO electrode using chronoamperometry at +1 V/SCE. (**B**) Contact angle of PPy/NiPc-SDS film electrodeposited on an FTO electrode using chronoamperometry at +1 V/SCE.

The PPy-SDS film displayed very hydrophilic behavior, with a water contact angle of less than 5°, while the PPy/NiPc-SDS film was more hydrophobic, with a contact angle between 50 and 70 °C (Figure 6). These measurements were in direct correlation with the film structure, not because of the presence of the surfactant, since the surfactant was present in both films, but due to the presence of the insoluble dye.

### 3.2. Development of Polymer-Based Microwave Sensors

- Fabrication and chemical modification of the microwave gas sensors

**Figure 7:**
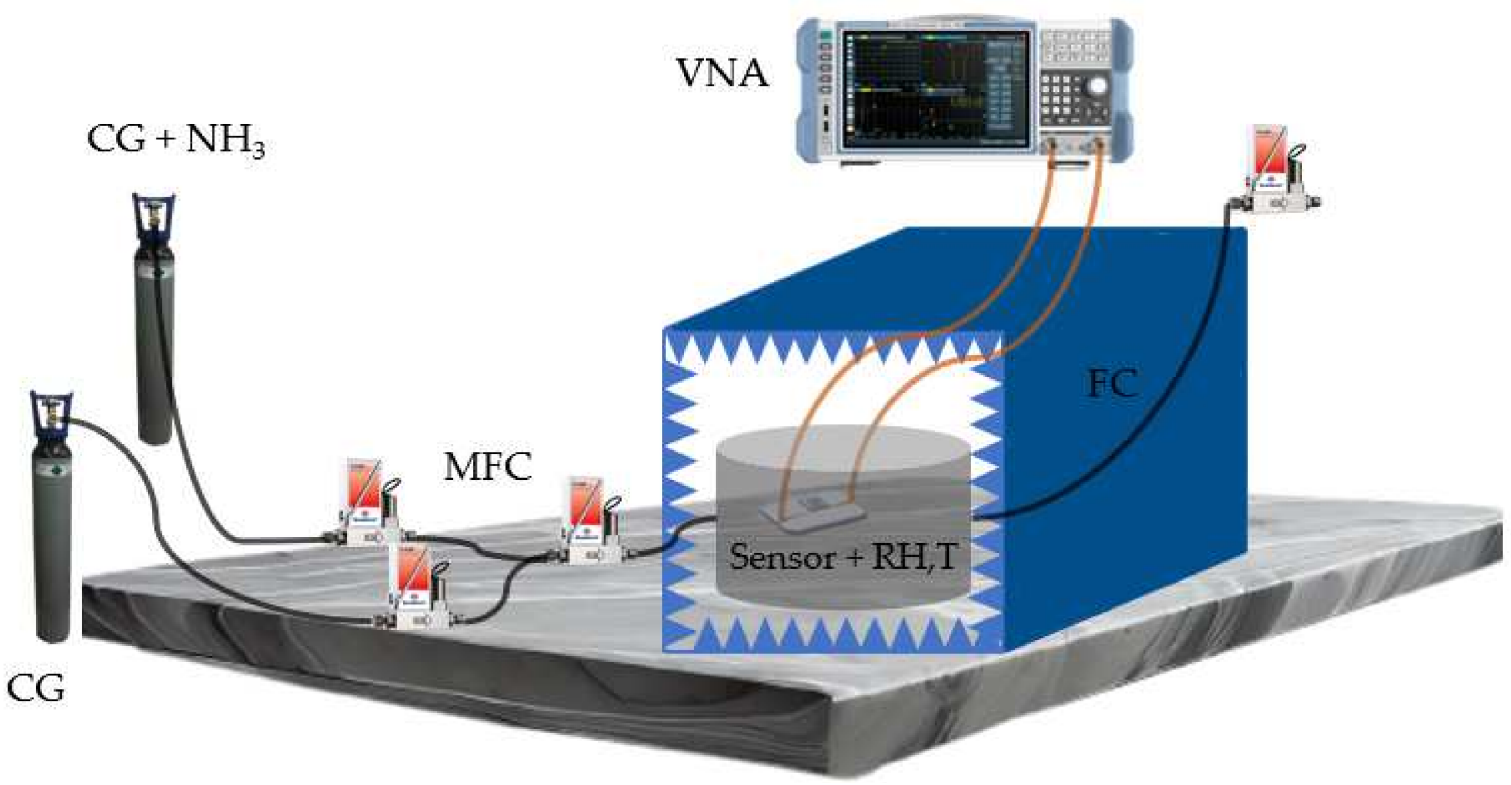
Setup that was used: VNA = Vector Network Analyzer, CG = carrier gas, MFC = mass flow controller, RH = relative humidity sensor, T = temperature sensor, FC = Faraday cage, MS = mass spectrometer [45].

Gas generation was ensured by calibrated Air Liquide gas cylinders (air and air + ammonia up to 400 ppm) with a guaranteed water concentration below 3 ppm. The gas circulated in the measuring bench through Teflon tubes, and the flow management was carried out using Bronkhorst EL Flow mass flow controllers. These devices also managed the dilution of the gas mixture (air and air + ammonia). The cell was either charged with the carrier gas alone (air) or with the carrier gas polluted with the target gas (air + ammonia). The switch from one to the other was made by activating a pneumatic valve. The cell was purged by sweeping with dry air. The cell filling time was estimated to be 30 *s*. The gas sensor was successively exposed to ammonia concentrations in air for 20 min, followed by dry air purges for 25 min. The hermetic cell was made entirely of glass to limit coadsorption phenomena. The sensor was connected via low-attenuation coaxial cables to a Rohde Schwarz R&D ZVB Vector Network Analyzer (VNA) that analyzed microwave variations over a range from 10 MHz to 20 GHz. The entire setup was controlled by a LabView interface on a dedicated computer. Figure 7 shows a schematic representation of the setup that was used.

**Figure 8:**
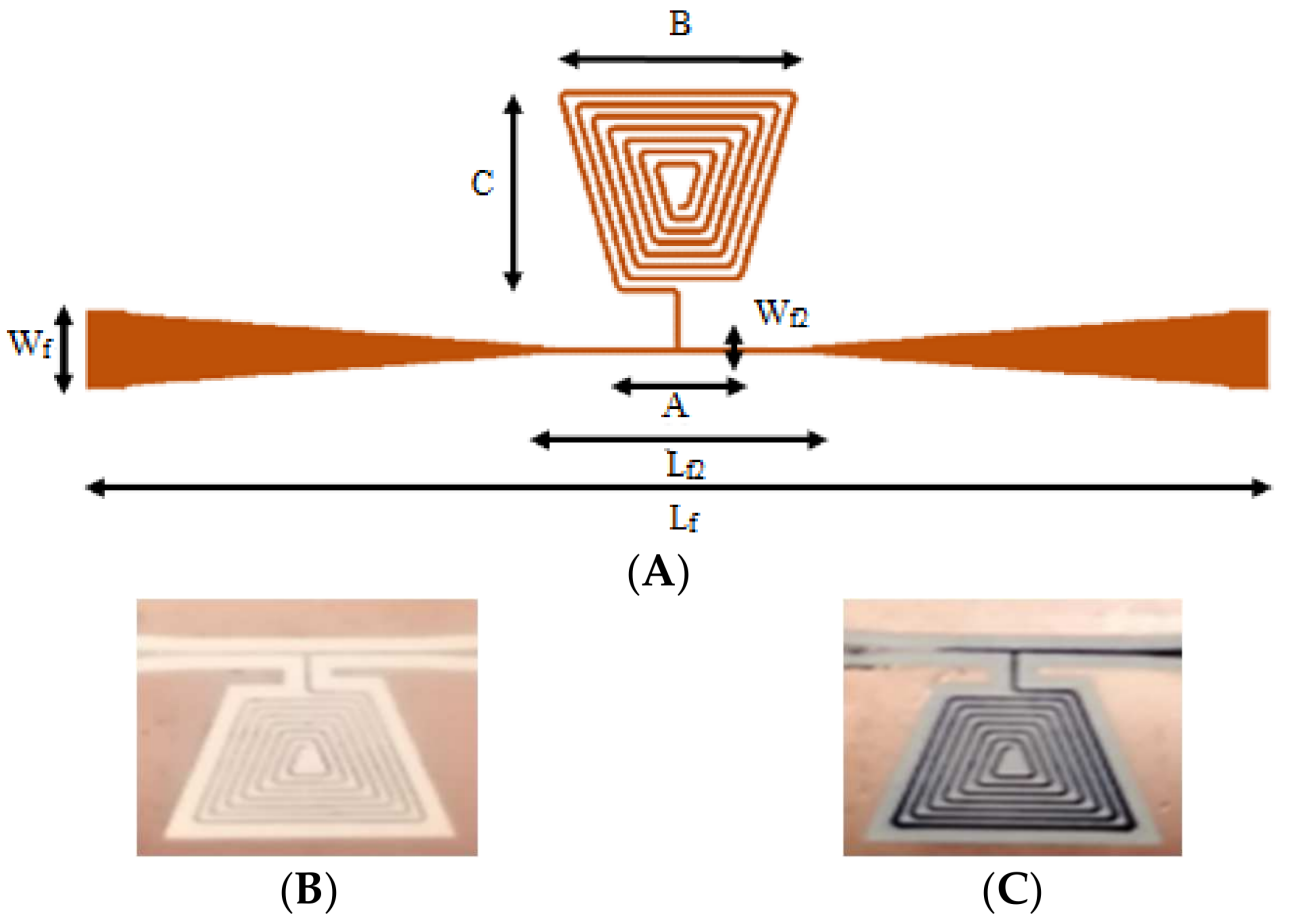
(**A**) Resonator structure and its dimensions (**A**): Wf = 2 mm, Wf2 = 0.150 mm, Lf = 30 mm, Lf2 = 6 mm, A = 3 mm, B = 6 mm, C = 5 mm, number of coils = 7, coils width = 0.150 mm, coil spacing = 0.150 mm [40]. (**B**) Gas sensor without deposited sensitive material. (**C**) Gas sensor coated with a PPy/NiPc-SDS sensitive layer (this layer was electrodeposited using chronoamperometry by applying a potential of +1.0 V/SCE for 5 min).

The sensor that was used was a microstrip spiral resonator with six resonant frequencies equally distributed in the range from 2 to 8 GHz [40]. The characteristics of the gas sensor are given in Figure 8A. A picture of a gas sensor without a deposited sensitive material is given in Figure 8B, as is a picture of a gas sensor coated with a PPy/NiPc-SDS film (Figure 8C) obtained using potentiostatic electrodeposition for 5 min at +1 V/SCE.

- Gas sensing results

Microwave measurements consist of generating incident waves with known parameters (magnitude, phase, frequency, etc.) via the VNA and transmitting them to the gas sensor via the coaxial cables. Some of the incident waves are reflected by the gas sensor, while the other waves are transmitted. The interaction between the surrounding gas and the sensitive material deposited on the circuit causes changes in physical and chemical properties such as the permittivity of the material. Thus, the principle of the measurement is to observe the microwave variations that are translated by the modification of the parameters of the reflected and transmitted waves compared to the incident waves. It is a kind of measurement of the reflection and transmission coefficient.

**Figure 9:**
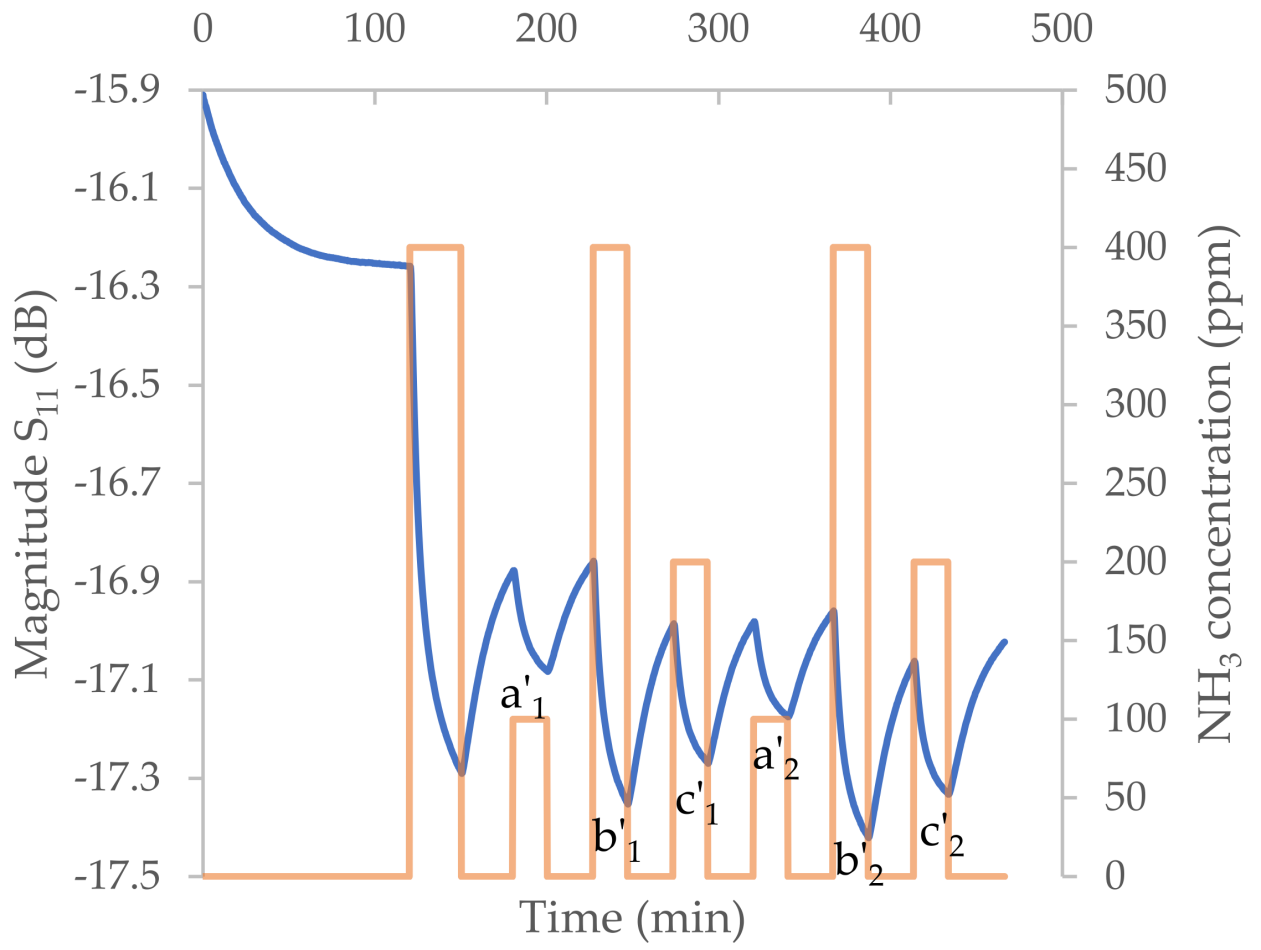
Variations in the magnitude of the PPy/NiPc−SDS microwave sensor (in blue) and the ammonia concentration in air (in orange) over time at 6.12 GHz.

**Figure 10:**
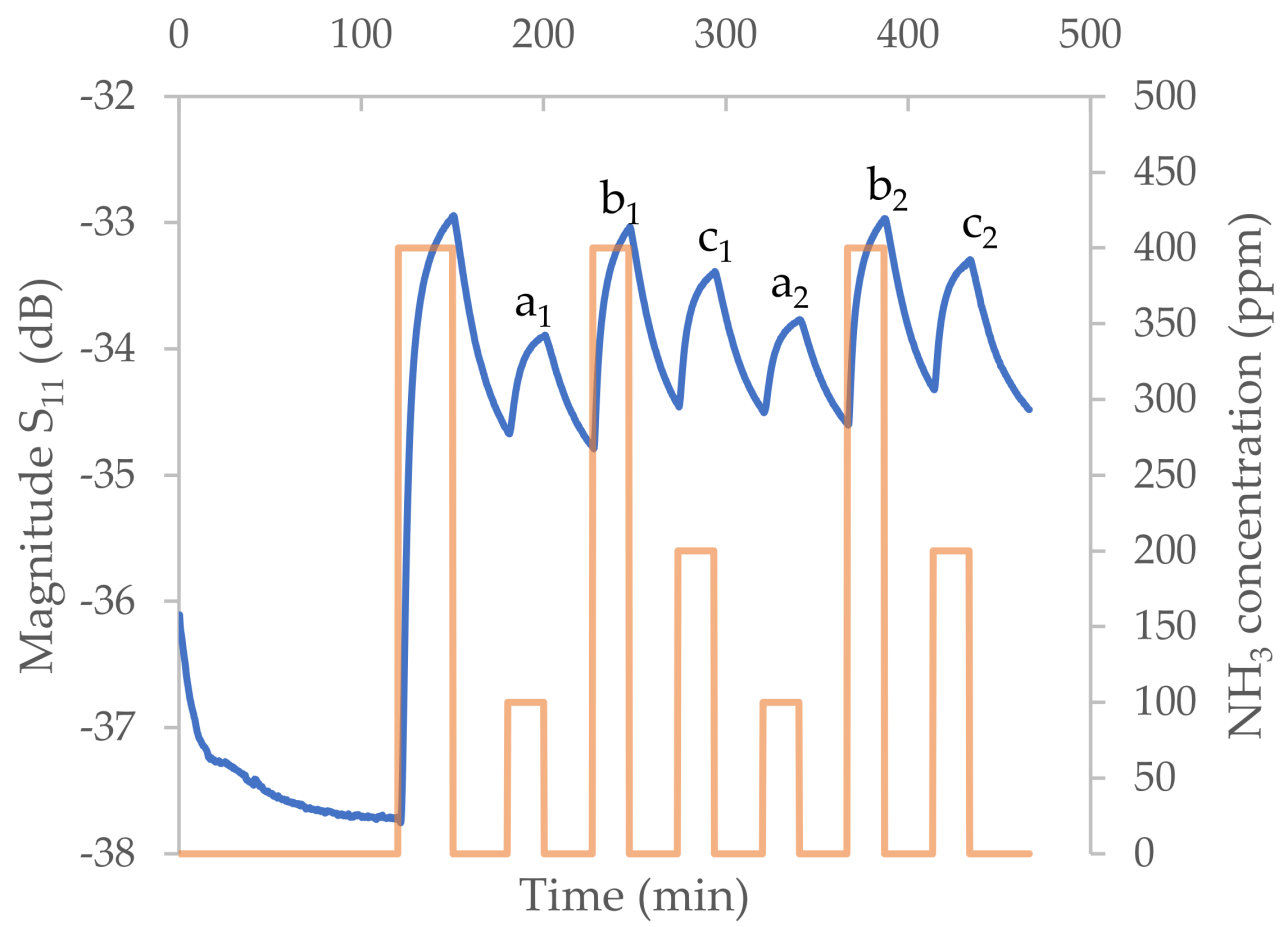
Variations in the magnitude of the PPy−SDS microwave sensor (in blue) and the ammonia concentration in air (in orange) over time at 6.75 GHz.

Figure 9 and Figure 10 show the microwave responses (magnitude, in blue) of the PPy/NiPc-SDS gas sensor and the PPy-SDS gas sensor and the ammonia concentration in air (in orange) as a function of time, respectively. A label is assigned to each ammonia exposure peak. For example, for the first exposure of the sensor to 100 ppm ammonia for PPy/NiPc-SDS film (Figure 9), the peak is defined as (a′_1_), and for the second exposure to 100 ppm ammonia for the same film, the peak is defined as (a′_2_). Respectively, these same exposure peaks are indicated by the labels (a_1_) and (a_2_) for the PPy-SDS film in Figure 10.

What should be observed here is not the absolute value of the magnitude or even the direction of variation but the value of the magnitude variation for each ammonia pulse at different concentrations. The value of the magnitude is not relevant because it depends on the analysis frequency. However, the two sensors did not have the same resonance frequencies since the deposited materials were not identical, so the analysis was not carried out at the same frequency. Moreover, it should be noted that in the case of the PPy/NiPc-SDS sensor the analysis was carried out at 6.12 GHz, while in the case of the PPy-SDS sensor it was carried out at 6.75 GHz, which corresponded to the frequency of the same resonance but had been shifted according to the nature of the electrodeposited polymer film. Thus, the direction of the magnitude variation did not reflect any physical or chemical phenomenon linked to the interaction of the target with the material but was simply dependent on whether the analysis was carried out right below or above the real resonance frequency of the sensor. Indeed, it was not possible to perform an analysis at the exact value of the resonance frequency since the measurements that were performed were broadband measurements and there was a finite number of experimental points over the chosen working frequency range. As a result, each analysis was performed at the experimental frequency closest to the system’s “real” resonant frequency to obtain the greatest variations in the microwave parameters. The choice of the experimental analysis frequency and the difference between this frequency and the sensor’s “real” resonance frequency were directly linked to the VNA’s resolution and parameter settings.

However, in both cases, the microwave response was considered to be qualitative and quantitative. The qualitative response meant that the variation in the microwave response was directly linked to the presence or the absence of ammonia in the air. For each increase or decrease in the target gas concentration, there was a quick variation in the magnitude. Moreover, the response time of the sensor was very short (a few seconds), making this device a real-time detection tool. This observation was consistent with the findings of previous work that highlighted the ability of the gas sensor to respond in real time to a change in the pollutant gas concentration. These observations were made and proven using a mass spectrometer directly connected to a gas circuit [45]. Furthermore, the quantitative aspect of the gas sensor response was highlighted by the fact that as the ammonia concentration in the air increased, the variation in the microwave response also increased. Both characteristics are essential for effective target detection. Finally, the response of the microwave sensor was particularly stable in the sense that the drift observed in the response was very low regardless of the sensor.

**Figure 11:**
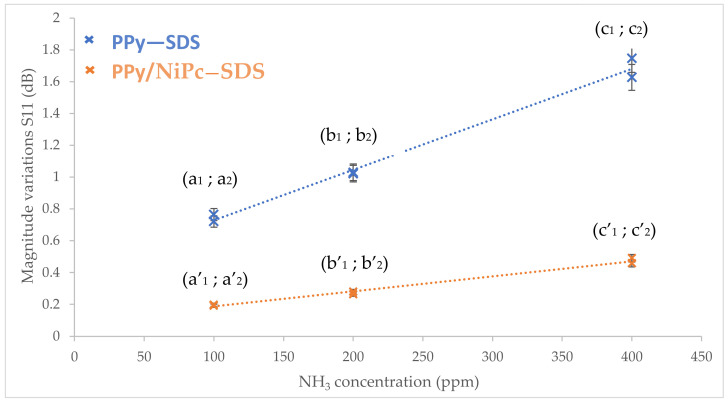
Variations in the magnitude of the microwave responses for the PPy−SDS sensor and the PPy/NiPc−SDS sensor as a function of the ammonia concentration in air.

Figure 11 shows the magnitude variation in the two gas sensors (blue—PPy-SDS and orange—PPy/NiPc-SDS) as a function of the NH_3_ concentration. The labels indicated (x,y) or (x’,y) correspond to the labels defined for Figure 9 and Figure 10. Figure 11 highlights the high stability of the signal at concentrations above 100 ppm. This is consistent with one of our previous papers that showed a more in-depth study of the stability of the microwave device over time [46]. Moreover, a linearization of the magnitude variations as a function of the ammonia concentrations in the air showed a linearity coefficient of about 99% for both sensors. This was consistent with the results of Wang et al., who demonstrated the existence of a linear or quasi-linear range of amplitude variations with respect to the ammonia concentration at concentrations above 50 ppm in air using a microwave sensor coated with a SnO_2_/bionic porous carbon composite material [47].

It can also be seen that the film without nickel phthalocyanine (PPy-SDS sensor) caused the greatest variation compared to the film containing it (PPy/NiPc-SDS sensor). The sensitivity of the PPy-SDS sensor was 3 to 4 times greater than that of the PPy/NiPc-SDS sensor. This was consistent with our expectations: the film of the PPy-SDS sensor was hydrophilic, whereas the film of the PPy/NiPc-SDS sensor was more hydrophobic. Thus, the effect of residual water (a major interferent) was much weaker in the case of the PPy/NiPc-SDS sensor. However, other work has shown that the main reason for the drift in microwave sensor responses is the uncontrolled presence of water [45]. Here, the conditions in the laboratory were carefully controlled, a precise protocol for conditioning the gas sensor was carried out before each experiment, the gas cylinders were calibrated and guaranteed a low concentration of water, and the cell was hermetically sealed so that water variations were non-existent or extremely low during the experiments.

As mentioned above, the estimated response time was very short, between 1.5 and 5 s, depending on the concentration. The repeatability of the PPy-SDS-coated sensor was estimated to be 5.5% for 100 ppm NH_3_, 5.4% for 200 ppm, and 7.3% for 400 ppm. The repeatability of the PPy-NiPc-SDS-coated sensor was very similar: 6.0% for 100 ppm NH_3_, 5.2% for 200 ppm, and 7.3% for 400 ppm. In addition, good stability and repeatability of the microwave device are prerequisites for machine learning using experimental data [46]. Although this type of work has not yet been carried out on the sensors presented in this paper, other work by the team has demonstrated the ability of a TiO_2_-coated microwave device to detect and quantify an ammonia concentration with 92% accuracy under conditions similar to those in this paper in the 0–400 ppm range using machine learning technology [45]. The sensitivity of the sensor in both cases was estimated from Figure 11. The PPy-SDS-film-coated sensor had a sensitivity of 3.2 mdB/ppm, while the PPy/NiPc-SDS-film-coated sensor had a sensitivity of 0.98 mdB/ppm. The sensitivity values given (as absolute values) are the result of averaging the sensitivities evaluated at 100, 200, and 400 ppm. Since a very high degree of linearity has been demonstrated in this concentration range, the sensitivities for these three concentrations were consistent with each other.

Compared with sensors already presented in the literature for the detection of ammonia using a hybrid layer, the presented sensors have fairly similar, if not better, characteristics in terms of response time (a few seconds, depending on the concentration), but this work has not shown that it can quantify or detect ammonia below the concentration that many sensors can detect today [48,49,50,51,52,53]. This is mainly due to the maturity of the original transduction used in this paper. Indeed, microwave transduction is relatively new compared with more conventional transductions, and certain technological hurdles are yet to be overcome. Microwave sensors are still at a stage of research where proof of concept is only focused on laboratory conditions. This corresponds to a TRL level well below that of most conventional transducers. However, one of the perspectives of this work and one of the objectives of our teams is to compare, in the next study, the detection quality of an industrial sensor and our home-made sensor. However, the results presented in this paper are very encouraging since, compared with other microwave sensors for ammonia in the literature, this sensor has very good characteristics, particularly in terms of sensitivity [45,46,47,54].

## 4. Conclusions

Solutions composed of pyrrole with and without nickel phthalocyanine were electro-oxidized in the presence of an anionic surfactant. This oxidation led to the formation of polypyrrole or polypyrrole/phthalocyanine films, as evidenced by infrared spectroscopy and energy-dispersive spectroscopy. The electrodeposited films showed similar cauliflower-like structures but very different wettability measurements. These films were then used as sensitive layers for microwave gas sensors. The resulting sensors were found to be sensitive to ammonia, and a linear relationship was established between the response of both films and the quantity of ammonia gas for concentrations above 100 ppm. In addition, the response under controlled laboratory conditions was greater for the more hydrophilic film (PPy-SDS) than for the hydrophobic film (PPy/NiPc-SDS). Although the PPy-SDS-coated sensor appeared to be more efficient at first, work carried out on the same sensor coated with another sensitive material (TiO_2_) suggested that this state is highly dependent on variations in relative humidity during measurement. Indeed, under outdoor measurement conditions, the relative humidity is constantly changing, and since the PPy-SDS sensor is hydrophilic, the drift in the signal from this sensor is expected to be much greater than the drift in the signal from the PPy/NiPC-SDS sensor, which is hydrophobic. These hypotheses will be the subjects of our future work and need to be backed up by precise data. In addition, a study of the sensor’s responses at lower concentrations (i.e., outside the range of linearity discussed above) will also be carried out. In both cases, and depending on the experimental conditions, the stability and sensitivity of the presented sensors demonstrate the benefits of combining microwave transduction and electrochemical polymer synthesis to overcome the difficulties currently encountered by other types of gas sensors.

## Figures and Tables

**Table 1:** Quantification results (weights) of PPy-SDS and PPy/NiPc-SDS films.

	W% (C)	W% (N)	W% (O)	W% (Na)	W% (S)	W% (Ni)
PPy-SDS	47.44	9.92	28.43	1.62	12.57	0
PPy/NiPc-SDS	45.24	8.13	26.56	6.11	13.50	0.45

## Data Availability

The data presented in this study are available on request from the corresponding author. The data are not publicly available due to privacy.
